# Forest-Stream Linkages: Effects of Terrestrial Invertebrate Input and Light on Diet and Growth of Brown Trout (*Salmo trutta*) in a Boreal Forest Stream

**DOI:** 10.1371/journal.pone.0036462

**Published:** 2012-05-04

**Authors:** Tibor Erős, Pär Gustafsson, Larry A. Greenberg, Eva Bergman

**Affiliations:** 1 Department of Biology, Karlstad University, Karlstad, Sweden; 2 Centre for Ecological Research, Balaton Limnological Institute of the Hungarian Academy of Sciences, Tihany, Hungary; 3 County Board of Värmland, Karlstad, Sweden; University of California, Berkeley, United States of America

## Abstract

Subsidies of energy and material from the riparian zone have large impacts on recipient stream habitats. Human-induced changes, such as deforestation, may profoundly affect these pathways. However, the strength of individual factors on stream ecosystems is poorly understood since the factors involved often interact in complex ways. We isolated two of these factors, manipulating the flux of terrestrial input and the intensity of light in a 2×2 factorial design, where we followed the growth and diet of two size-classes of brown trout (*Salmo trutta*) and the development of periphyton, grazer macroinvertebrates, terrestrial invertebrate inputs, and drift in twelve 20 m long enclosed stream reaches in a five-month-long experiment in a boreal coniferous forest stream. We found that light intensity, which was artificially increased 2.5 times above ambient levels, had an effect on grazer density, but no detectable effect on chlorophyll *a* biomass. We also found a seasonal effect on the amount of drift and that the reduction of terrestrial prey input, accomplished by covering enclosures with transparent plastic, had a negative impact on the amount of terrestrial invertebrates in the drift. Further, trout growth was strongly seasonal and followed the same pattern as drift biomass, and the reduction of terrestrial prey input had a negative effect on trout growth. Diet analysis was consistent with growth differences, showing that trout in open enclosures consumed relatively more terrestrial prey in summer than trout living in covered enclosures. We also predicted ontogenetic differences in the diet and growth of old and young trout, where we expected old fish to be more affected by the terrestrial prey reduction, but we found little evidence of ontogenetic differences. Overall, our results showed that reduced terrestrial prey inputs, as would be expected from forest harvesting, shaped differences in the growth and diet of the top predator, brown trout.

## Introduction

Forests adjacent to streams and rivers potentially have a great impact on the aquatic community as forests not only provide a substantial energy base for the lotic communities, i.e. FPOM/CPOM, woody debris and invertebrates, but also influence solar radiation, flow regime, nutrient runoff and temperature. As small streams have a high surface area to volume ratio and a high degree of shading these are particularly affected by surrounding forests [1],[2]. Thus, modifications of the riparian vegetation, as occurs, for example, during forest harvesting, may have profound effects on stream biota. This has become of increasing concern since the 1950s, when more mechanized forestry practices started [3] in [4]. As a consequence of highly mechanized and large-scale forestry practices, riparian zones have become greatly modified, thereby altering the pathways for energy flux from the terrestrial to the aquatic habitats, with consequences for stream-dwelling fish [4],[5].

Modern forestry practices have changed over the last 40 years, with a growing environmental awareness, where the use of harvesting practices that entail clear-cutting down to the stream edge has decreased [4]. However, even in developed countries there is still a lack of understanding for the importance of saving broad bands of riparian vegetation. In Sweden, for example, there is no law forcing landowners to retain riparian vegetation, only recommendations from the Swedish Forest Agency [6]. During the last 10 years there has been an increase in the number of forest fellings that have paid little attention to these recommendations [6]. There are numerous field studies showing that clear-cutting affects, for example, sedimentation, nutrient runoff, solar illumination and insect production [4], which in turn may affect the aquatic habitat and fish fauna. However, as these studies evaluate the simultaneous effect of several variables that are important for aquatic habitats and stream-dwelling fish [4], it is hard to evaluate the specific effect of any one single factor. Two factors that have the potential to affect stream-dwelling fish such as brown trout (*Salmo trutta*) are light and terrestrial invertebrate input. These factors affect forest-dependent energy pathways to streams, and the strength of them depends on both the extent and structure of riparian tree vegetation [2],[7],[8]. Primary production in forested streams is believed to be limited by incoming solar radiation (e.g. [9]) due to shading by tree canopies. Light usually affects stream food webs via bottom-up processes, and the general consensus is that increased incident light is positively correlated with increased primary production [9]–[11] and thus might favor increased abundance of grazers, a potential prey for stream-dwelling fish. That terrestrial invertebrate subsidies are an important food resource for stream-dwelling fish was first noticed by Allen [12], and since then numerous studies have shown strong interconnections between the terrestrial falling insects and the performance of drift foraging fish populations in forested streams [13]–[20].

Parallel with studies reporting that forested riparian zones generally have higher inputs of falling terrestrial invertebrates than unforested riparian zones [21],[22], other descriptive studies have reported that during some parts of the year terrestrial invertebrates are frequently consumed by salmonids and may contribute >50% of total food intake [13]–[20],[23]. Terrestrial invertebrates may be beneficial for stream fish for several reasons. Firstly, the input of terrestrial invertebrates normally peaks during summer, corresponding to a period when aquatic benthic invertebrates are often in short supply and energy demands in fish are high due to high water temperatures [24]–[26]. Secondly, terrestrial invertebrates typically fall into streams during daytime when aquatic drift may be low. This, in combination with a generally high buoyancy and large size compared to aquatic macroinvertebrates, makes terrestrial invertebrates a conspicuous and highly energetically profitable prey for a silhouette-feeding fish ([16],[19] and references therein, [27]). Thirdly, although somewhat debated, terrestrial invertebrates may also be more nutritious than aquatic invertebrates [2],[28]. The documented use and assumed advantages of terrestrial invertebrates represent a direct link between trees and trout, where a reduction of the terrestrial resource may have a negative effect on trout.

The factors influenced by the structure and composition of the riparian zone are rarely permanent in their ability to drive stream ecosystem processes; instead they often show seasonal variation. For example, the flux of terrestrial inputs into streams is seasonally variable [2],[29], with high inputs of terrestrial invertebrates often occurring in summer when the availability of suitable aquatic benthic macroinvertebrates is low [24],[26]. As fish energetic requirements are high at this time [25], a reduction in terrestrial prey supply may have its largest impact on fish populations and trophic interactions during summer. Moreover, there is a general consensus that seasonal changes in light availability can be traced in patterns of primary production, although the seasonal changes in light, temperature, nutrients and herbivore abundance also may affect primary production in complex ways, making it difficult to generalize bottom-up effects in stream ecosystems [9], [30]–[32]. Thus, the understanding of stream function is challenged by seasonal variation in autochthonous production and abundance of different organisms.

Temporal variability in a resource subsidy such as terrestrial invertebrates may have population level consequences, affecting for example fitness, growth or intraspecific competition [33]–[35]. Examination of population level effects may reveal rather straight-forward effects on patterns of growth, diet and habitat use for a population. However, as many animal populations are size-structured, whereby individuals undergo ontogenetic niche shifts [36]–[40], temporal variation in resource subsidies may also affect size-classes in a population differently. For example, some studies have observed that the diet of large trout contain higher proportions of terrestrial invertebrates than the diet of small trout [41], [42]. As terrestrial invertebrates are generally larger in size than their aquatic counterparts [22],[43], the differences in diet may reflect an ontogenetic niche shift. Such size-dependency may have both direct and indirect effects on the outcome of trophic level interactions.

Light level and terrestrial subsidies represent two important factors that link streams with their surrounding forests, and both of these factors are affected by forest harvesting. Consequently, we wanted to manipulate these two factors to isolate how two factors associated with forest harvesting affect stream biota. Here, we examine the effects of increased light levels and reduced terrestrial invertebrate input on a brown trout population in a small coniferous forest stream. Specifically, we examined the 1) growth and diet of two size classes of brown trout (*Salmo trutta*), 2) the biomass of benthic algae, 3) the density of invertebrate grazers, and 4) the potential food resources of brown trout, i.e. composition and biomass of drift and terrestrial invertebrate input. This was done in a five-month-long field enclosure experiment. Our overall hypothesis was that trout growth and diet would be affected by both increased light level and reduced terrestrial invertebrate input. More specifically we hypothesized that increased light level would increase biomass of benthic algae or grazer abundance, which in turn would affect the diet and growth of brown trout. We also hypothesized that reduced input of terrestrial invertebrates would 1. lead to a decreased amount of terrestrial prey in the drift, 2. induce decreased trout growth rates and that these reductions would show ontogenetic differences, with larger trout being more influenced than smaller due to size-dependent foraging strategies, and 3. vary over the season, having its largest impact on trout growth during summer when terrestrial invertebrate inputs were expected to peak.

## Materials and Methods

### Ethics Statement

Great care was taken in handling the fish throughout this study to minimize any negative effects on the fish. This includes electrofishing, PIT-tagging and sampling routines such as weighing, length measuring and stomach flushing. This study was carried out in accordance with current laws and ethical concerns in Sweden, being approved (Certificate number: 92-2006) by the Gothenburg Ethical Committee.

### Study site and experimental treatments

The experiment was conducted in a ∼700 m long section of Sundtjärnsbäcken, a first order lake outlet stream situated in the Glaskogen Forest Reserve in central Sweden (X-Y coordinates; 6612710-1303106). The stream is a typical forest stream for the region, with a stony, gravel bottom and an average width of 1–1.5 m. The riparian forest provides a dense canopy over the stream (>70%) and is dominated by coniferous *Picea abies* and *Pinus sylvestris*, although there are some deciduous species present such as *Betula pendula* and *Alnus glutinosa*. The stream is oligotrophic (NO_3_ <100 µg/l; NO_2_ <1 µg/l; NH_4_ <10 µg/l; PO_4_-P <1 µg/l; P-total<6 µg/l), with a pH of 6.9–7.8. Brown trout (*Salmo trutta*) is the only resident fish in the stream. It should be noted that the year prior to the experiment (2005) a severe drought extirpated most of the fish population. Before starting the experiment the stream was electrofished to ensure that no fish were present in the experimental area.

**Figure 1:**
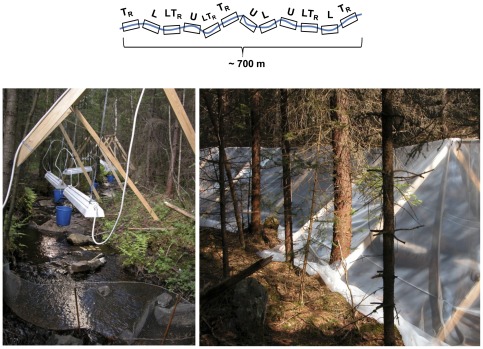
Illustration and photograph of the experimental area. Schematic sketch of the stream and the distribution of the four treatments (T_R_, L, T_R_L and U) and their replicates. Enclosures were 20 m long and separated from each other by a minimum of 40-m long undisturbed buffer sections. Photographs show a L and a T_R_ enclosure.

Terrestrial prey input and light were manipulated in a 2×2 factorial design with three replicates per treatment. The four treatments were: (1) Unmanipulated (referred to as U: ambient light levels and terrestrial inputs, (2) Terrestrial input reduction (T_R_: reduced terrestrial prey input and ambient light level), (3) Light enhancement (L: natural light conditions, which were supplemented with artificial light, and ambient terrestrial input) and finally (4) Reduced terrestrial input and enhanced light level applied together (T_R_L). The replicates of each treatment were semi-randomly distributed, with the stipulation that replicates of the same treatment could not be placed adjacent to each other. As a result the replicates of the different treatments were well distributed along the entire length of the stream (Fig. 1). The 12 experimental enclosures were 20 m long, producing enclosure areas of 20–30 m^2^, which is approximately the home range reported for juvenile brown trout [44]. The enclosures were separated from each other by 40-m long buffer sections. The enclosures were established using 7.5-mm wire mesh fences, and the mesh size represented a compromise between retaining the fish and creating an environment with moderate current velocities, allowing most invertebrates to pass through the nets, e.g. [45]–[47]. The nets were buried 15–20 cm into the stream bottom and supported with gravel to prevent fish escaping. To prevent clogging of the nets, they were cleaned 1–3 times per week, depending on weather and season.

To reduce terrestrial invertebrate input, 2.5 m high plastic tents supported by wooden frames were built over the entire T_R_ and T_R_L enclosures. The tent covers were made of UV–resistant, 95%-transparent greenhouse plastic, attached to both the wooden frames and the ground floor. To ensure that drifting terrestrial input would be low, an additional 20 m long tent section was built upstream each T_R_ and T_R_L enclosure. Each end of the tent enclosure was covered with mosquito-netting, with a small opening near the top to reduce influx of aerial insects, but allowing emerging insects from within the tent to leave. In addition, eight 40×15 cm openings were cut into the roof of the tent to allow insects to leave the covered enclosures. To control for the physical presence of the wooden frames, we put up the same type of frames over the U and L enclosures.

To increase light levels, we used 8 lamp houses (150×30×10 cm) equipped with two fluorescent tubes (TL-D 840, 58 W, 1200×30 mm) per lamp in each enclosure. The fluorescent tubes imitate photosynthetic active radiation with wavelengths mainly between 400 and 700 nm. The lamp houses were hung, c. 50 cm apart and 60 cm above the stream bottom, from the wooden frames. Originally the lamps were hung about 40 cm from the stream bottom but due to spring floods at the end of May the lamps had to be raised 20 cm. By doing so the amount of PAR (photosynthetic active radiation) reaching the water surface decreased from about 65 to 45–50 µmol m^−2^ s^−1^. To control for the physical presence of the lamps, we suspended lamp models in the T_R_ and U enclosures. This was done using white plastic boards of the same size as the lamp houses, which were hung from wooden frames in the same way as in the L and T_R_L treatments. The lamps (i.e. light) were programmed via electronic timers providing a daily light/dark photoperiod. To avoid undesired attraction of terrestrial invertebrates to the lamps the timers were set to switch on the light one hour after dawn and switch off one hour before dusk. To correct for changes in day length over seasons all timers were manually re-programmed on a weekly basis.

**Figure 2:**
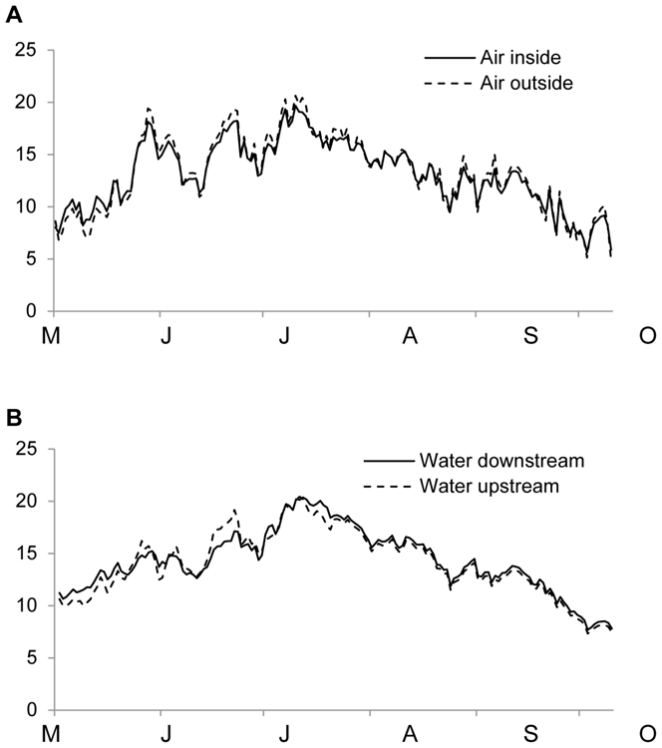
Air and water temperature. Downstream (continuous line) and upstream (dashed line) daily mean air (A) and water (B) temperature (°C) in Sundtjärnsbäcken Creek during the five month study. Error bars have been omitted for clarity.

Mean daily air temperature inside and outside one of the tents, as measured with two data loggers, was similar (Fig. 2). Similarly, mean daily water temperature at the most upstream and downstream enclosures were nearly the same (Fig. 2). Physical habitat data, measured at the onset of the experiment, did not show any differences among treatments (Table 1), which indicates that the treatments did not differ in any of the physical factors generally known to influence the organization of stream biota.

**Table 1:** Basic data at the start of the experiment.

	T_R_			L			T_R_L			U		
	N	mean	SE	N	mean	SE	N	mean	SE	N	Mean	SE
Width (cm)	3	136	24	3	131	8	3	128	13	3	134	6
Depth (cm)	3	11	1	3	13	1	3	14	1	3	15	3
Velocity (cm s^−2^)	3	30	6	3	25	5	3	24	6	3	17	3
Sand (%)	3	14.4	7.3	3	10.7	8.6	3	26.7	7.2	3	25.6	9.1
Gravel (%)	3	29.6	5.5	3	19.6	8.7	3	42.6	8.7	3	28.1	9.3
Stone (%)	3	46.3	8.4	3	38.5	15.8	3	22.6	8.5	3	30.7	11.3
Rock (%)	3	9.6	3.7	3	31.1	14.9	3	8.1	8.1	3	15.6	6.8
SL-Y (mm)	36	79	1	37	80	1	36	80	1	35	79	1
SL-O (mm)	17	135	6	17	137	4	18	139	5	18	138	6
W-Y (g)	36	4.7	0.2	37	4.7	0.2	36	4.8	0.2	35	4.7	0.2
W-O (g)	17	23.3	3.0	17	23.1	2.1	18	25.1	3.3	18	24.0	3.0
Density (ind m^−2^)	3	0.69	0.12	3	0.69	0.04	3	0.72	0.08	3	0.66	0.04

Basic physical habitat characteristics and fish data (mean ± 1 SE) in the different treatments at the start of the experiment. U = unmanipulated, L = light enhancement, T_R_ = terrestrial input reduction, T_R_L = T_R_ and L applied simultaneously. SL denotes standard length, whereas W indicates weight for young (Y) and old (O) fish. Separate univariate ANOVAs analyses were performed for all variables and none were significant (P>0.3 for all comparisons).

The fish used in the experiment were electrofished in a nearby stream and anaesthetized with MS-222, measured for total length (mm) and weight (0.01 g) and individually marked with PIT-tags (TROVAN ID-100). They were held for 24 h before stocking them into the enclosures on 17 May 2006 at a pre-calculated density of ∼0.70 trout m^−2^, which was well within the range found in similar streams in the surrounding area. Two size groups, hereafter referred to as young and old fish, were used to represent the natural population structure and to examine the size-dependent effects of terrestrial prey on trout growth and diet. Young and old fish had a mean length of 79 (±0.6 SE) and 137 (±3) mm and a mean biomass of 4.7 (±0.1) and 23.9 (±1.41) g, respectively. Altogether 11–13 young and 5–6 old fish were translocated to each enclosure, depending on the area of the enclosure and the size of the fish. Separate one-way analyses of variance (ANOVA) did not reveal differences among the treatments for length, weight and density variables for young or for old fish (P>0.3 in each case; see Table 1).

### Sampling and laboratory analyses

Sampling occurred on five occasions: 10–11 May, 19–20 June, 1–2 August, 2–3 September and 8–9 October. However, terrestrial invertebrate input was also sampled once between each of these occasions (i.e. totally nine occasions) and light levels (PAR) were measured weekly.

Input of terrestrial invertebrates was measured using bucket-traps suspended over the stream. Six traps (51 cm^2^ per trap) were used in each enclosure, spaced to proportionally sample the whole enclosure area. The bottom of the traps was covered with approximately 2–3 cm of water together with a few drops of detergent. After 72 h the content of the traps was sieved with a 500-µm net and preserved in 70% ethanol. All samples were treated separately for later laboratory analyses.

Photosynthetic active radiation (PAR, 400–700 nm, µmol m^−2^ s^−1^) was measured between 10:00 and 14:00 h under a range of weather conditions using a light meter (LI-18, Leiderdorp Instruments). At each sampling, nine measurements were taken directly above the water surface in each of the 12 enclosures. These were taken at two meter intervals along the entire length of the enclosure, alternately from the left side, middle and right side of the enclosures.

Three weeks before the start of the experiment 350 glazed ceramic tiles (5×5 cm) were incubated in the stream. Although unglazed tiles typically have higher colonization rates than glazed tiles [48], grazing effects have been observed using glazed tiles [46]. On 15 May, 30 tiles were placed in each enclosure, with ten tiles placed in the lower, middle and upper third of each enclosure. On each sampling occasion two tiles from each of the three locations in each enclosure (i.e. 6 tiles in each enclosure) were randomly selected for analysis of chlorophyll *a* concentrations. We only took tiles without sediment and the tiles were individually placed in plastic boxes, wrapped in aluminum folia and frozen within half an hour. In the laboratory, chlorophyll *a* content was analyzed using methanol extraction with subsequent spectrophotometrical analysis (SS 28 170).

Invertebrates drifting into the enclosures were sampled at the upstream end of each enclosure using a drift net (WILDCO, 40×30 cm). The nets were deployed twice for 60 min, in the morning and right after sunset. Drift density (g m^−3^) was calculated from measurements of water velocity (Höntzsh μP-flowtherm) at the center of each net, the width of the net and the mean water depth at the sampling spot. Drift samples were immediately preserved in 70% ethanol and treated separately for subsequent laboratory examinations. Because drift density was low on most sampling occasions, day and night drift data were later pooled to give a more robust estimation.

Benthic grazers were collected using a Surber sampler (20×20 cm). Six samples were collected from randomly selected positions in each enclosure on each occasion. The samples were sorted in the field and preserved in 70% ethanol and treated separately for laboratory examination. Benthic grazers were determined at the highest taxonomic resolution possible [49]. The biomass of all invertebrate taxa, including falling terrestrial, drift and instream collected animals, was obtained through the construction of taxon-specific length-weight regressions based on the individual length and weight (i.e. dry mass after drying at 60 °C for 24 h; [16]) from representative specimens from all taxa collected.

Fish were collected with a single pass, or occasionally, with two-pass electrofishing, resulting in 100, 94, 77, 89 and 66% of the fish being sampled on the different sampling occasions. Captured fish were anaesthetized with MS-222 and measured for standard length (mm) and body mass (0.01 g). Stomach contents were obtained from all captured fish by stomach flushing, which is an effective and non-lethal method to examine the diet of salmonids [50]. The stomach content of each fish was preserved in 70% ethanol and kept separately for laboratory analyses. The fish were then returned to their original enclosures. A few fish died during sampling and these were substituted with new PIT-tagged fish of similar size, so as to maintain density/biomass relations. We found only one dead fish in the stream enclosures during the entire study, and considering the high capture success based mostly on single pass electrofishing, we feel confident that mortality was low. In addition, due to a severe spring flood on 25–26 May many fish escaped from the enclosures. Within one to two days after the flood the escaped fish were recaptured and translocated back to their original enclosures. Although this accident could have affected the growth data for this time period, we have decided to show growth data for this time period so as to 1) show the complete data set of this long term experiment and 2) follow changes in seasonal growth, independent of treatment effects, which can be important in understanding brown trout ecology. Individual growth for each time period was calculated as specific growth rate (SGR, [51]) as:
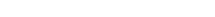
where W_1_ is the initial weight (g) at time t_1_, and W_2_ is the weight measured at time t_2_.

### Statistical analysis

We used separate repeated measures two factorial ANOVAs with time as the repeated measure and terrestrial prey (reduction vs. no reduction) and light (enhanced vs. ambient condition) as categorical predictors, to test the effects of these factors on chlorophyll *a* content, grazer abundance, drift biomass, proportion of terrestrial drift, and specific growth rate of trout. Because growth rates of trout differ between age groups (i.e. younger fish grow faster), we ran separate repeated measures ANOVAs for the two age groups. All variables had to be log (x+1) transformed before the analyses to satisfy assumptions of normality and reduce heteroscedasticity, except the proportion of terrestrial drift and proportion of terrestrial prey in the diet, which were arcsine square root transformed. Analyses were carried out with either the program STATISTICA or SPSS version 18.0–19.0.

Due to empty stomachs, sample sizes for old fish were too low to perform a repeated measure ANOVA on diets for the entire data set. Instead we investigated differences in trout diet with canonical analysis of principal coordinates (hereafter CAP). This recently developed constrained ordination method is identical to a non-parametric multivariate analysis of variance [52], [53]. Diet data from each sampling occasion were analyzed in separate analyses. However, the empty stomachs of old fish, which were most numerous in the light treatments, limited our ability to test both treatment effects in a single analysis. Therefore, we did not investigate the effect of light on diet composition; instead we tested the effects of the terrestrial input manipulation [reduction vs. no reduction] and age of fish [young vs. old]. Between 44 and 65 prey items (taxa) were identified during the four samplings. Of these, the ten most “abundant" taxa, comprising between 63 and 96% of the diet together, were selected for analyses of individual taxa based on the product of the relative frequency of occurrence and relative biomass of each taxon in the diet. The other items, which each comprised only a small proportion of the diet but were important when taken together, were pooled into three groups: 1) other aquatic, 2) other terrestrial, 3) adult flying aquatic insects. Consequently, each CAP analysis was based on 13 taxa (groups) (Table S1) as explanatory variables and 4 *a priori* defined groups (reduction and no reduction treatments for the two age classes). The CAP analyses were run based on relative biomass data, using Hellinger distances [54] and were carried out with the program CAP [55].

## Results

**Figure 3:**
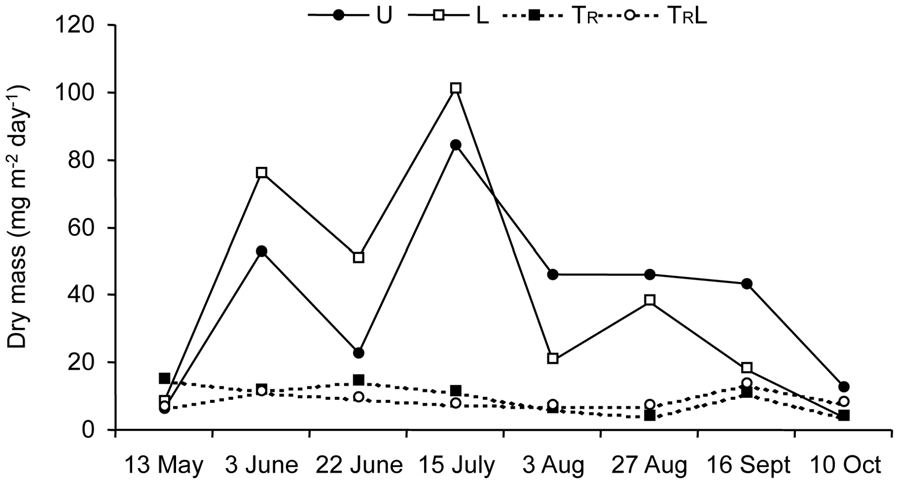
Flux of falling invertebrates. Changes in the flux of falling terrestrial invertebrates (mean dry mass, mg m^−2^ day^−1^) in unmanipulated (U), light (L), terrestrial invertebrate reduction (T_R_) ), terrestrial invertebrate reduction×light (T_R_L) treatments throughout the study. Error bars have been omitted for clarity.

The flux of terrestrial prey input showed large temporal variation in the uncovered enclosures, with a maximum input in July (Fig. 3), which contrasts with the relatively constant and low input in the covered enclosures. On average, uncovered enclosures received over four times more invertebrate biomass from May to October than covered enclosures, with an overall mean (± SE) of 39.5 (±7.2) and 39.4 (±9.6) mg m^−2^ d^−1^ for U and L, and 9.4 (±1.2) and 8.5 (±1.0) mg m^−2^ d^−1^ for T_R_ and T_R_L sections, respectively. Repeated measures ANOVA on the total biomass of these prey indicated significant effects of time (F_7,56_ = 8.42; P<0.001), terrestrial input reduction (F_1,8_ = 167.62; P<0.001) and a time×terrestrial input reduction interaction (F_7,56_ = 5.31; P<0.001), whereas the effects of light and associated interactions were not significant. In terms of taxonomic composition, Diptera, Araneida, Coleoptera and Hymenoptera were the dominant taxa, ranging from 73.3 to 80.6% of the total biomass among the four treatments. Flying aquatic insects were of relatively minor importance, comprising <10% of the biomass.

**Figure 4:**
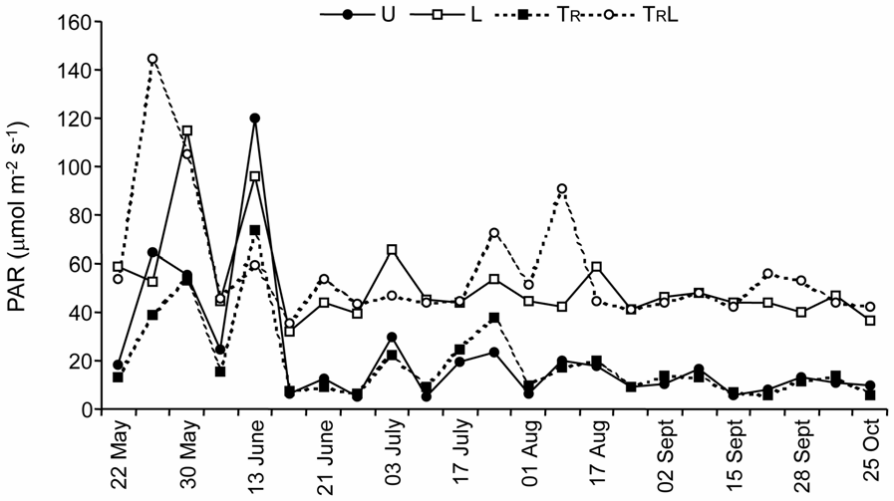
Light level in enclosures. Mean PAR (400–700 nm) values measured in unmanipulated (U), light (L), terrestrial invertebrate reduction (T_R_), terrestrial invertebrate reduction×light (T_R_L) treatments throughout the study period. Error bars have been omitted for clarity.

Light intensity varied substantially during the experiment, particularly early on (prior to mid-June) when foliage was developing (Fig. 4). Mean PAR values for U, T_R_, L and T_R_L treatments were 25.6±2.6 (SE), 22.8±1.7, 55.5±2.2 and 59.8±3.0 µmol m^−2^ s^−1^, respectively. Repeated measures ANOVA indicated a significant effect of time (F_22,176_ = 8.96; P<0.001), light (F_1,8_ = 46.54; P<0.001) and a time×light interaction (F_22,176_ = 3.27; P<0.001), whereas other effects were not significant.

**Figure 5:**
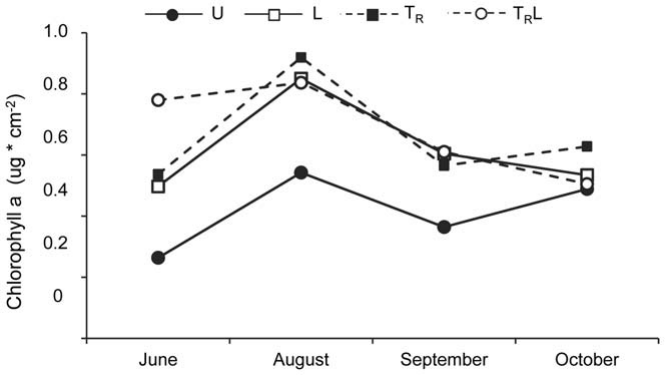
Chlorophyll a. Chlorophyll a (ug/cm^2^) over the whole season in unmanipulated (U), light (L), terrestrial invertebrate reduction (T_R_), terrestrial invertebrate reduction×light (T_R_L) treatments. Error bars have been omitted for clarity.

Mean chlorophyll *a* biomass varied between 0.26±0.17 (SE) µg cm^−2^ (U in June) and 0.91±0.03 (T_R_ in August) µg cm^−2^ during the experiment (Fig. 5). However, repeated measures ANOVA did not reveal any effects of time (F_3,24_ = 2.0, P = 0.14), light (F _1,8_ = 1.4, P = 0.28), terrestrial input reduction (F_1,8_ = 3.1, P = 0.12) or their interactions (P>0.38 for the different interactions).

**Figure 6:**
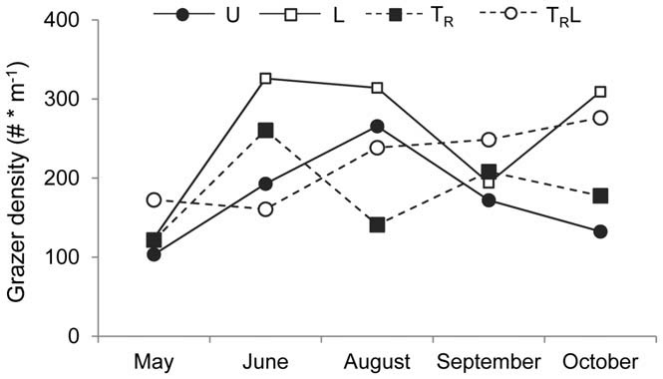
Grazer density. Grazer density (mean number ± SE per m^2^) over the whole season in unmanipulated (U), light (L), terrestrial invertebrate reduction (T_R_), terrestrial invertebrate reduction×light (T_R_L) treatments. Error bars have been omitted for clarity.

The grazers were dominated by Orthocladinae, *Elmis* larvae and adult, *Oulimnius* larvae and adult, and *Nemoura* (25.3%, 19.2%, 15.9% and 12.3% by numbers, respectively). The number of grazers varied between 103±46 (SE) (U in May) and 326±65 (L in June) and grazer biomass between 0.019±0.009 g/m^2^ (T in October) and 0.057±0.019 g/m^2^ (T in June) during the experiment (Fig. 6). Repeated measures ANOVA based on number of grazers revealed an effect of time (F_4, 32_ = 4.268; P = 0.007) and light (F_1, 8_ = 5.811, P = 0.042) but no effects of terrestrial input reduction (F_1, 8_ = 0.0001, P = 0.983) or any of their interactions (P>0.3). A similar analysis based on biomass showed no significant effects of time, light, terrestrial input reduction or any of their interactions (P>0.2), probably due to the relatively few but heavy molluscs masking any biomass effect of other taxa.

**Figure 7:**
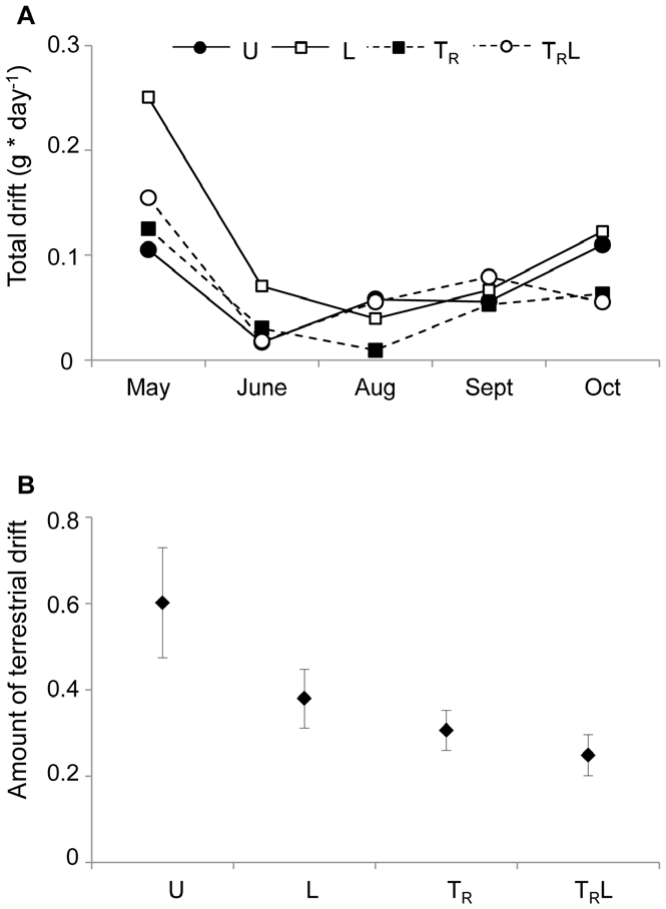
Total and terrestrial drift. Total drift (g/day, mean ± SE) over the whole season (A) and amount of terrestrial organisms in the drift (biomass based %, seasonal mean ± SE) (B) in unmanipulated (U), light (L), terrestrial invertebrate reduction (T_R_), terrestrial invertebrate reduction×light (T_R_L) treatments.

Based on biomass, the aquatic part of the drift consisted mainly of Simuliidae, Coleoptera and Plecoptera (19.7%, 14.1%, 12.8%, respectively), whereas the terrestrial part was dominated by Hymenoptera, Diptera, Coleoptera and Araneae (37%, 27%, 19%, 7.5% respectively). Drift abundance showed a strong temporal pattern (Fig. 7A), with a maximum value in May, largely due to the high biomass of aquatic invertebrates and a minimum in June and August. Repeated measures ANOVA on total biomass revealed an effect of time (F_4,32_ = 4.84; P = 0.004) but no effects of light (F_1,8_ = 2.6, P = 0.14), terrestrial reduction (F_1,8_ = 2.0, P = 0.19) or interactions (P>0.5 for the different interactions). However, repeated measures ANOVA on the proportion of terrestrial drift showed an effect of terrestrial reduction (F_1,8_ = 8.265, P = 0.021), but no effect of time (F_4,32_ = 0.872; P = 0.491), light (F_1,8_ = 3.74, P = 0.089) or any of the interactions (P>0.2 for the different interactions). Enclosures with terrestrial reduction had a lower proportion of terrestrial organisms in the drift than other enclosures (Fig. 7B).

**Figure 8:**
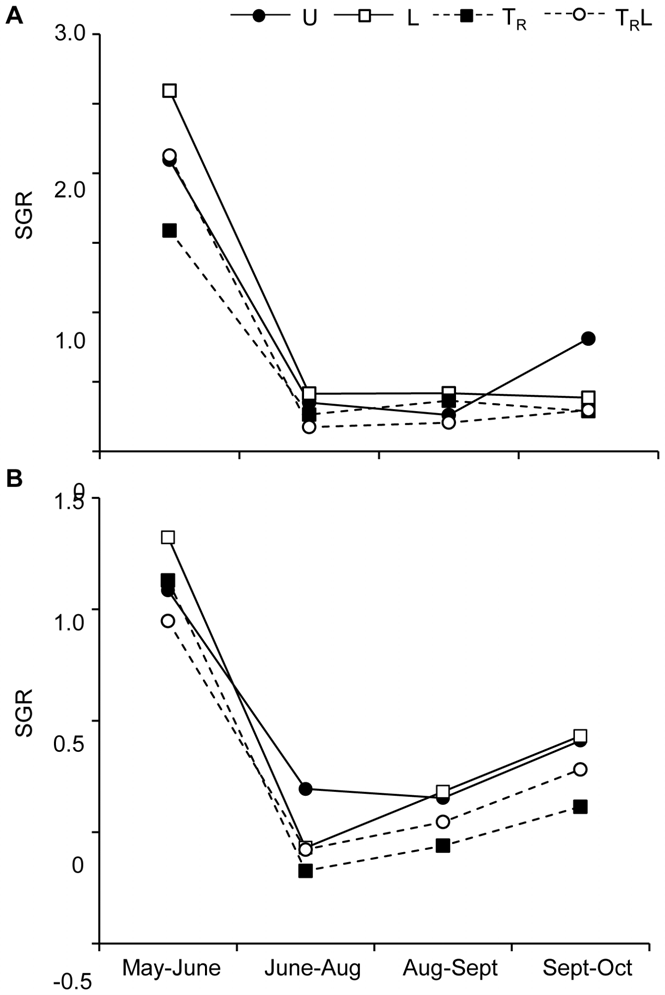
Trout specific growth rate. The specific growth rate (SGR; biomass data [% per day]) of young (A) and old (B) trout in unmanipulated (U), light (L), terrestrial invertebrate reduction (T_R_) and terrestrial invertebrate reduction×light (T_R_L) treatments during the four study periods. Note that scale of the y-axis differs between size classes. Error bars have been omitted for clarity.

Growth rates of both young and old trout were, just as total drift biomass, highest in May–June, with mean values of 2.2% day^−1^ for young fish and 1.1% day^−1^ for old fish (Fig. 8). During subsequent periods, growth was substantially lower, typically <0.5% day^−1^ and in some cases negative growth rates were observed for old fish (Fig. 8B). Repeated measures ANOVAs of growth rates for old fish indicated a significant effect of terrestrial input reduction and time, but not for light or any of the interactions (Table 2). For young fish there was a significant effect of time and time×terrestrial input reduction×light treatment interaction (Table 2). Because data for young fish were difficult to interpret due to the interaction, two-way ANOVAs were performed separately for each sampling period. The results showed significantly lower growth rates for young due to terrestrial input reduction effects for the May–June, June–August and September–October period (F_1, 98_ = 12.9, P = 0.0005 for May–June, F_1,90_ = 5.9, P = 0. 017 for June–August, F_1, 83_ = 6.32, P = 0.014), but not for the August–September period (F_1, 77_ = 0.15, P = 0.698). There was also a significant effect of light in May–June for young fish (F_1, 98_ = 14.5, P = 0.0002). Consequently, the effect of terrestrial reduction varied among time periods for young trout, whereas it was consistent over time for old trout.

**Table 2:** Two factor ANOVA testing for the effect on trout growth.

	Young				Old			
Source	df	MS	F	P	df	MS	F	P
Terrestrial input	1	0.034	2.936	0.098	1	0.133	8.085	***0.011***
Light	1	0.003	0.259	0.615	1	0.014	0.831	0.374
Terrestrial input×Light	1	0.000	0.006	0.939	1	0.021	1.248	0.279
Error	27	0.012			18	0.016		
Time	3	0.889	122.332	***<0.001***	3	0.577	45.777	***<0.001***
Time×Terrestrial input	3	0.007	0.979	0.407	3	0.026	2.024	0.121
Time×Light	3	0.010	1.424	0.242	3	0.008	0.613	0.610
Time×Terrestrial input×Light	3	0.028	3.799	***0.013***	3	0.008	0.643	0.591
Error	81	0.007			54	0.013		

Summary table of repeated measures two factor ANOVA testing for the effect of light and terrestrial invertebrate input on the seasonal growth of young and old trout.

Diet of trout contained a broad diversity of prey of both aquatic and terrestrial origin. In total 79 taxa were identified in the diet during the whole season. The most common aquatic prey were chironomid larvae and pupae, *Simulium* larvae and Dysticidae larvae, and the most common terrestrial prey were Lumbricidae, adult terrestrial dipterans and Arachnidae. All of these prey items occurred in 15% or more of the analyzed stomachs.

**Figure 9:**
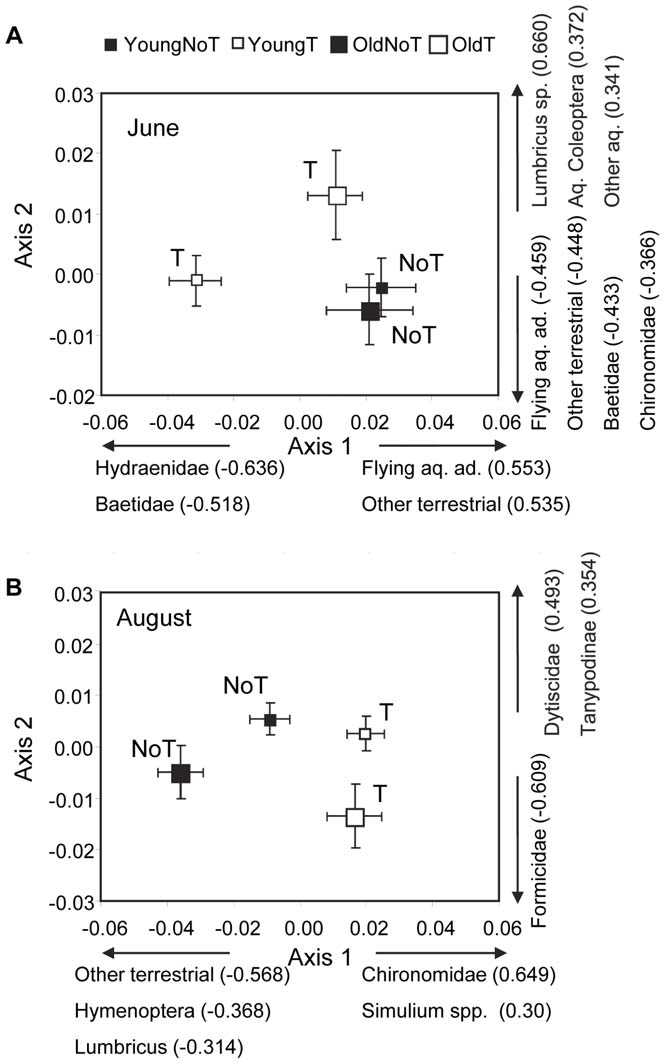
Trout diet. Canonical analysis of principal coordinates of diet composition data in (A) June and (B) August. Mean axis scores with SE values are shown for each treatment type (Young and Old indicates young and old fish, respectively, whereas T and NoT indicates terrestrial invertebrate reduction and no terrestrial invertebrate reduction treatments). Food items showing axis correlations ≥0.3 (Spearman's rho in parenthesis; P<0.05 in all cases) are indicated for each axis.

The CAP analysis was performed on 13 prey taxa/groups, based on the rank order abundance for each monthly sample (22 taxa altogether, Table S1). The overall trace statistics of the canonical analysis of principal coordinates indicated significant dietary differences between the four treatments (i.e. diet of young and old fish in enclosures with reduced terrestrial input (“covered") vs. enclosures with ambient terrestrial input (“open")) for June, August and September but not for October (Table 3). For the first two axes from the CAP analysis, the first axis for both months separated dietary items largely based on terrestrial vs. aquatic origin of prey, and treatment differences were significant only for June and August. In June young trout had a significantly higher consumption of small aquatic prey (mainly Hydraenide and Baetide) in the covered enclosures than young and old trout in the other three treatments, where fish from these three treatments had a higher consumption of flying aquatic organisms and prey of terrestrial origin (Fig. 9). In August there were greater treatment differences in trout diet than in June, and the samples from the covered and open enclosures were well separated along the first ordination axis (Fig. 9). Both young and old trout had a relatively higher proportion of “other terrestrial taxa", Hymenoptera and *Lumbricus spp* in the open enclosures than in the covered enclosures, where instead Chironomidae and *Simulium spp* larvae dominated the diet (Fig. 9). There was also a tendency for large trout to eat more ants than small trout as seen by the age class separation along the second axis (Fig. 9).

**Table 3:** Results of the CAP analysis of diet composition.

Month	M	Proportion of SS_T_	Trace statistics	P	δ1^2^	P
June	5	0.716	0.308	0.027	0.239	0.009
August	10	0.951	0.330	0.032	0.237	0.004
September	12	0.991	0.448	0.018	0.206	0.136
October	4	0.698	0.160	0.701	0.125	0.546

Summary results of the CAP analysis of diet composition data for young and old brown trout during the four periods of the experiment. “M" is the number of principal coordinate (PCO) axes involved in the final CAP analysis. (Note that this is chosen by the program itself, based on minimizing the residual or misclassification error.) “Proportion of SS_T_" is the number of the total sum of squared interpoint dissimilarities divided by the number of points (total variance) explained by the first “m" PCO axes. “δ_1_^2^" is the canonical correlation value for the first axis. For details on CAP, see [52].

## Discussion

Our results suggest the changes in light and terrestrial input, as would be expected from forestry practices that involve clear-cutting of large areas close to streams, can affect stream-dwelling fish such as brown trout [4]. Our manipulation of light levels showed no effect on the primary producer level, but instead there was an effect on grazer abundance, indicating a bottom-up response. Further, our manipulation of across-habitat resource subsidies, e. g. terrestrial inputs, showed effects on drift composition, trout diet and trout growth. Moreover, the effects on trout growth and diet were influenced by both fish size and season, but the size-dependent effects were smaller than expected.

We had expected that our light manipulated enclosures, via bottom-up effects, would have had a positive effect on fish growth, especially towards the end of the experiment. Instead, we found almost no effect of light on fish growth. The only significant effect on growth occurred in the May–June period for young fish. At this time total drift was higher, but not significantly higher, in the light manipulated enclosures, and this higher drift may explain why there was an effect of light on fish growth. Even if drift was higher in illuminated enclosures, a growth difference at the beginning of the experiment cannot easily be ascribed to bottom-up effects as too little time had elapsed.

We had also expected an effect of light on the development of periphyton (i.e. chlorophyll *a* biomass), but such an effect was not observed. Instead, we found a higher abundance of grazers in our enclosures with increased light levels, which is consistent with studies showing that increased periphyton productivity may become directly transformed into grazer biomass [9], [30], [56]. Such rapid transformation is further supported by evidence of grazing as indicated by the tracks of snails on the tiles in our study (members of the family Planorbidae, Valvatidae, Hydrobiidae and Elobiidae were found in the stream).

Alternative explanations to the lack of an increase in periphyton (chlorophyll a) biomass to the increased light levels could be related to that the absolute light levels in the manipulated enclosures were too low, or that the stream was nutrient-limited instead of light-limited. Photosynthetic-irradiance measurements suggest that photosynthesis by stream periphyton is saturated between 100 and 200 µmol m^−2^ s^−1^ [1], and that the photosynthetic rate of shade-adapted algae can substantially increase from 20–25 (as our U and T_R_ enclosures) to 55–60 µmol m^−2^ s^−1^ (as our L and T_R_L enclosures)(see e.g., Fig. 2. in [9]). This suggests that our light increase could support an increase in periphyton production. However, the interactions between irradiance, nutrients and herbivores are complex, making it difficult to predict the outcome of light manipulations [9], . Sundstjärnsbäcken is oligotrophic, with total phosphorous levels around 6 µg/l [58]–[60]. The experimental design does not allow us to rule out that light and/or nutrient levels were too low to affect periphyton. Nevertheless, we believe that the lack of periphyton biomass increase was most likely caused by a bottom-up effect, i.e. transforming periphyton biomass into the observed increase in grazer abundance.

We had expected that there would be ontogenetic differences in the growth and diet of trout in response to reductions of terrestrial subsidies. This is because numerous field studies have shown that there is a positive correlation between body size and the relative contribution of surface-drifting prey to the diet of salmonids, [40]–[42], [61]. Moreover, large, dominant salmonids inhabit pools [62]–[64] and forage in the water column, where they encounter and use generally large and conspicuous surface-drifting terrestrial invertebrates [19], [22], [43]. In contrast small fish spend most of their time in riffles [61], [65]–[67], where they forage mainly on benthic prey [42], [68]. Our study showed that there was an overall effect of reduced terrestrial subsidies on the growth of old trout, whereas for young trout there was an effect for three of the four sampling periods, i.e. no effect in August–September. During the first two of these sampling periods there was also high terrestrial inputs in uncovered enclosures (Fig. 3). This indicates that there was only a small difference in the growth response of old and young trout.

The diet of trout also showed small differences between young and old trout. In June the diet of old trout was dominated by terrestrial and flying aquatic adult prey in all enclosures, with and without reduced terrestrial subsidies, whereas the diet of young trout was dominated by aquatic prey when terrestrial subsidies were reduced. In August the diet of large trout, and to a somewhat smaller extent the diet of small trout, was dominated by terrestrial prey items only in enclosures with unmanipulated terrestrial subsidies. The response by both age classes in August could be a response to the overall low availability of terrestrial invertebrates at this time. Thus, our results on growth and diet indicate that the expected effects of across-habitat resource subsidies on populations were strong, but ontogenetic differences were not as strong as we expected.

Although explored in the field (Nakano & Murakami 2001), the relationship between seasonally variable within-habitat food resources and across-habitat resource subsidies has not received much experimental attention. In streams aquatic invertebrates are typically abundant in spring and early summer, followed by a reduction in availability, largely due to the emergence of many aquatic invertebrate taxa [17], [23], [24], [26]. Thus, during summer the aquatic invertebrates present in the stream, although often numerous, are small in size and not particularly suitable as food for trout. Our study showed more or less the same pattern for drifting animals, with a relatively high drift biomass in May, followed by lower drift biomass during the summer and fall. The influx of terrestrial invertebrates has also been shown to vary seasonally [2], [29]. In our study, the influx of terrestrial invertebrates was greatest in summer and thus should have been able to compensate to some extent for the low availability of aquatic invertebrates at this time [24], [26].

Given that both aquatic resources and terrestrial subsidies vary seasonally, the growth of salmonids in the recipient habitat is also expected to vary seasonally [69]–[71]. This was the case in Sundtjärnsbäcken as the growth of trout was highest in spring, followed by a reduction in summer, and a slight increase again in autumn. Such temporal patterns in growth rate thus correspond well to the seasonal dynamics of drifting invertebrates, which have a maximum in spring [16], [23], [24], [26]. The high influx of terrestrial subsidies in summer compensates to some degree for the low availability of aquatic invertebrates at this time, but growth rates were nowhere near those observed in May. This is presumably because of the high metabolic demands of brown trout during the warm summer months, with temperatures reaching 20°C (Fig. 1), which is suboptimal for trout growth [25]. Thus, these results indicate that the impact of across-habitat subsidies on the recipient habitat is not only dependent on availability of aquatic and terrestrial prey, but also on the physiological constraints of the animals living in the recipient habitat.

The present study indicates that there is an effect of terrestrial input on trout growth and such effects have not been experimentally examined previously in coniferous forest streams. Furthermore, our study indicates that the effects may be similar to those reported from deciduous forest streams [16], [34], even though terrestrial invertebrate inputs in coniferous forests are generally believed to be lower than in their deciduous counterparts [72]. Our study also indicates there may be a bottom-up effect of light, suggesting that light might be a regulator in autotrophic food chains in forested oligotrophic boreal streams. We found that terrestrial prey subsidies may play a substantial role in the growth and diet of a top fish predator and highlights how the strength of this interaction depends on season but much less strongly on fish size. Forest managers must thus realize that their actions may not only influence the forest itself, but also the structure and function of the streams flowing through these forests.

## Supporting Information

Table S1 **Trout diet.** Seasonal changes in the frequency of occurrence (O%) and mean relative biomass (A%) of the most important food items in the diet of young (Y) and old (O) trout in unmanipulated (U), light (L), terrestrial invertebrate reduction (T_R_) and terrestrial invertebrate reduction and light (T_R_L) treatments throughout the study period. Chironomidae* refers to all taxa except Tanypodinae.
(DOCX)
