# Supplementary material for: Forest-Stream Linkages: Effects of Terrestrial Invertebrate Input and Light on Diet and Growth of Brown Trout (Salmo trutta) in a Boreal Forest Stream
Source: PLoS One. 2012 May 4;7(5):e36462. doi: 10.1371/journal.pone.0036462 (PMC3344874; doi:10.1371/journal.pone.0036462)
Supplement: Table S1 — Trout diet. Seasonal changes in the frequency of occurrence (O%) and mean relative biomass (A%) of the most important food items in the diet of young (Y) and old (O) trout in unmanipulated (U), light (L), terrestrial invertebrate reduction (TR) and terrestrial invertebrate reduction and light (TRL) treatments throughout the study period. Chironomidae* refers to all taxa except Tanypodinae. (DOCX) [file pone.0036462.s001.docx]

**Table S1**. Seasonal changes in the frequency of occurrence (O%) and mean relative biomass (A%) of the most important food items in the diet of young (Y) and old (O) trout in unmanipulated (U), light (L), terrestrial invertebrate reduction (T_R_) and terrestrial invertebrate reduction and light (T_R_L) treatments throughout the study period. Chironomidae* refers to all taxa except Tanypodinae.

| June taxa | YC | | YL | | YT | | YTL | | OC | | OL | | OT | | OTL | |
| --- | --- | --- | --- | --- | --- | --- | --- | --- | --- | --- | --- | --- | --- | --- | --- | --- |
|  | O% | A% | O% | A% | O% | A% | O% | A% | O% | A% | O% | A% | O% | A% | O% | A% |
| *Lumbricus sp.* | 17,65 | 11,66 | 10,00 | 2,04 | 20,00 | 14,87 | 7,14 | 1,34 | 0,00 | 0,00 | 0,00 | 0,00 | 16,67 | 6,35 | 50,00 | 28,11 |
| Chironomidae* | 100,00 | 22,60 | 100,00 | 15,98 | 75,00 | 12,00 | 92,86 | 33,89 | 90,91 | 33,60 | 75,00 | 41,63 | 116,67 | 24,30 | 100,00 | 24,83 |
| Coleoptera (aquatic, adult) | 5,88 | 0,45 | 20,00 | 3,44 | 5,00 | 1,20 | 7,14 | 1,37 | 0,00 | 0,00 | 25,00 | 13,48 | 33,33 | 19,27 | 37,50 | 3,44 |
| Araneida | 0,00 | 0,00 | 50,00 | 15,37 | 10,00 | 1,64 | 0,00 | 0,00 | 27,27 | 2,14 | 50,00 | 12,15 | 50,00 | 6,30 | 0,00 | 0,00 |
| Nemouridae larvae | 23,53 | 6,65 | 40,00 | 4,36 | 35,00 | 8,20 | 28,57 | 8,70 | 27,27 | 1,84 | 0,00 | 0,00 | 50,00 | 5,45 | 37,50 | 1,32 |
| Heteroptera | 0,00 | 0,00 | 30,00 | 0,09 | 10,00 | 3,69 | 0,00 | 0,00 | 0,00 | 0,00 | 25,00 | 8,42 | 16,67 | 0,27 | 0,00 | 0,00 |
| Baetidae larvae | 35,29 | 6,99 | 30,00 | 0,92 | 50,00 | 11,82 | 50,00 | 16,39 | 54,55 | 10,51 | 0,00 | 0,00 | 50,00 | 3,10 | 37,50 | 2,06 |
| Dytiscidae (adult) | 17,65 | 1,57 | 60,00 | 8,14 | 20,00 | 5,09 | 35,71 | 4,13 | 27,27 | 2,11 | 25,00 | 0,25 | 33,33 | 0,97 | 0,00 | 0,00 |
| *Hydraena spp.* (adult) | 5,88 | 4,45 | 10,00 | 5,29 | 45,00 | 12,54 | 28,57 | 10,72 | 27,27 | 2,11 | 0,00 | 0,00 | 16,67 | 1,50 | 37,50 | 0,92 |
| *Simulium spp.* | 0,00 | 0,00 | 30,00 | 7,16 | 25,00 | 5,28 | 21,43 | 1,42 | 27,27 | 2,41 | 25,00 | 0,16 | 33,33 | 2,68 | 0,00 | 0,00 |
| Other aquatic | 41,18 | 12,63 | 70,00 | 29,78 | 75,00 | 17,77 | 50,00 | 15,75 | 63,64 | 21,31 | 25,00 | 3,12 | 83,33 | 15,09 | 62,50 | 34,86 |
| Other terrestrial | 64,71 | 14,34 | 60,00 | 3,15 | 45,00 | 2,69 | 21,43 | 2,82 | 45,45 | 6,42 | 25,00 | 10,39 | 50,00 | 7,36 | 50,00 | 2,06 |
| Flying aquatic adult | 64,71 | 18,66 | 60,00 | 4,28 | 45,00 | 3,20 | 21,43 | 3,47 | 54,55 | 17,54 | 25,00 | 10,39 | 50,00 | 7,36 | 50,00 | 2,39 |
| No. of fish with diet | 17,00 | | 10,00 | | 20,00 | | 14,00 | | 11,00 | | 4,00 | | 7,00 | | 8,00 | |

| August taxa | YC | | YL | | YT | | YTL | | OC | | OL | | OT | | OTL | |
| --- | --- | --- | --- | --- | --- | --- | --- | --- | --- | --- | --- | --- | --- | --- | --- | --- |
|  | O% | A% | O% | A% | O% | A% | O% | A% | O% | A% | O% | A% | O% | A% | O% | A% |
| Chironomidae* | 72,73 | 22,85 | 73,91 | 21,58 | 79,31 | 40,32 | 70,59 | 25,84 | 58,33 | 7,48 | 27,27 | 1,40 | 75,00 | 20,00 | 81,82 | 36,02 |
| *Lumbricus sp.* | 0,00 | 0,00 | 17,39 | 15,41 | 0,00 | 0,00 | 5,88 | 5,76 | 0,00 | 0,00 | 27,27 | 24,98 | 0,00 | 0,00 | 9,09 | 8,55 |
| Hymenoptera | 13,64 | 3,23 | 17,39 | 3,37 | 10,34 | 0,37 | 11,76 | 5,78 | 8,33 | 0,49 | 36,36 | 22,41 | 12,50 | 2,84 | 18,18 | 0,73 |
| Araneida | 9,09 | 4,20 | 13,04 | 5,67 | 0,00 | 0,00 | 17,65 | 9,75 | 25,00 | 13,14 | 0,00 | 0,00 | 25,00 | 5,64 | 0,00 | 0,00 |
| *Simulium spp.* | 36,36 | 10,72 | 26,09 | 6,95 | 41,38 | 17,03 | 58,82 | 12,69 | 25,00 | 5,06 | 27,27 | 6,80 | 37,50 | 15,94 | 45,45 | 8,50 |
| Dytiscidae (adult) | 4,55 | 2,13 | 21,74 | 6,17 | 13,79 | 4,44 | 11,76 | 1,50 | 8,33 | 2,46 | 9,09 | 2,19 | 0,00 | 0,00 | 0,00 | 0,00 |
| Formicidae | 0,00 | 0,00 | 8,70 | 4,28 | 10,34 | 4,79 | 5,88 | 0,27 | 16,67 | 3,36 | 0,00 | 0,00 | 12,50 | 8,70 | 18,18 | 15,65 |
| Tanypodinae larvae | 36,36 | 15,23 | 34,78 | 10,23 | 37,93 | 9,38 | 23,53 | 7,25 | 25,00 | 12,60 | 9,09 | 9,09 | 37,50 | 5,37 | 36,36 | 2,03 |
| Chironomidae pupae | 18,18 | 5,13 | 17,39 | 3,76 | 6,90 | 3,63 | 5,88 | 5,39 | 25,00 | 5,63 | 18,18 | 5,89 | 25,00 | 8,63 | 27,27 | 0,94 |
| Homoptera | 4,55 | 0,21 | 0,00 | 0,00 | 6,90 | 0,22 | 5,88 | 0,67 | 25,00 | 12,56 | 0,00 | 0,00 | 0,00 | 0,00 | 0,00 | 0,00 |
| Other aquatic | 31,82 | 15,25 | 43,48 | 7,38 | 34,48 | 16,16 | 35,29 | 19,97 | 33,33 | 14,07 | 27,27 | 16,24 | 25,00 | 18,78 | 36,36 | 25,07 |
| Other terrestrial | 31,82 | 16,21 | 43,48 | 13,20 | 13,79 | 3,39 | 17,65 | 4,99 | 66,67 | 23,15 | 27,27 | 10,17 | 37,50 | 9,30 | 9,09 | 0,19 |
| Flying aquatic adult | 9,09 | 4,83 | 8,70 | 2,01 | 10,34 | 0,26 | 5,88 | 0,13 | 0,00 | 0,00 | 9,09 | 0,83 | 12,50 | 4,81 | 9,09 | 2,32 |
| No. of fish with diet | 22,00 | | 23,00 | | 29,00 | | 17,00 | | 12,00 | | 11,00 | | 8,00 | | 11,00 | |

| September taxa | YC | | YL | | YT | | YTL | | OC | | OL | | OT | | OTL | |
| --- | --- | --- | --- | --- | --- | --- | --- | --- | --- | --- | --- | --- | --- | --- | --- | --- |
|  | O% | A% | O% | A% | O% | A% | O% | A% | O% | A% | O% | A% | O% | A% | O% | A% |
| *Lumbricus sp.* | 33,33 | 26,12 | 25,00 | 17,16 | 25,00 | 20,43 | 13,04 | 9,02 | 30,77 | 9,21 | 45,45 | 26,38 | 0,00 | 0,00 | 12,50 | 8,90 |
| Lepidoptera larvae | 12,50 | 7,31 | 10,71 | 5,09 | 8,33 | 0,55 | 8,70 | 4,27 | 23,08 | 18,11 | 27,27 | 12,80 | 0,00 | 0,00 | 12,50 | 0,03 |
| *Velia sp.* | 25,00 | 12,26 | 21,43 | 6,02 | 8,33 | 0,49 | 13,04 | 4,76 | 15,38 | 2,20 | 9,09 | 2,55 | 0,00 | 0,00 | 25,00 | 18,18 |
| *Simulium spp.* | 25,00 | 12,58 | 53,57 | 10,81 | 66,67 | 8,37 | 26,09 | 8,65 | 7,69 | 0,56 | 54,55 | 5,53 | 12,50 | 2,14 | 12,50 | 0,77 |
| Chironomidae* | 66,67 | 5,33 | 57,14 | 9,36 | 83,33 | 23,55 | 56,52 | 11,38 | 53,85 | 5,99 | 54,55 | 1,39 | 50,00 | 23,20 | 62,50 | 23,34 |
| Araneida | 4,17 | 2,70 | 17,86 | 3,65 | 8,33 | 1,43 | 8,70 | 7,59 | 23,08 | 7,97 | 18,18 | 0,29 | 12,50 | 10,35 | 12,50 | 11,11 |
| Limoniidae larvae | 12,50 | 1,02 | 7,14 | 3,02 | 16,67 | 5,31 | 17,39 | 8,15 | 0,00 | 0,00 | 27,27 | 5,36 | 0,00 | 0,00 | 0,00 | 0,00 |
| Formicidae | 8,33 | 1,37 | 10,71 | 3,54 | 8,33 | 0,14 | 13,04 | 4,79 | 7,69 | 1,72 | 27,27 | 1,10 | 0,00 | 0,00 | 25,00 | 10,51 |
| Planorbidae | 0,00 | 0,00 | 7,14 | 3,99 | 0,00 | 0,00 | 4,35 | 3,40 | 0,00 | 0,00 | 18,18 | 11,91 | 0,00 | 0,00 | 12,50 | 9,69 |
| Limnephilidae larvae | 12,50 | 3,03 | 32,14 | 2,86 | 25,00 | 5,18 | 4,35 | 0,29 | 23,08 | 0,22 | 36,36 | 7,25 | 0,00 | 0,00 | 0,00 | 0,00 |
| Other aquatic | 75,00 | 19,39 | 85,71 | 28,66 | 83,33 | 31,44 | 82,61 | 33,51 | 84,62 | 30,87 | 100,00 | 20,14 | 62,50 | 57,55 | 75,00 | 7,67 |
| Other terrestrial | 37,50 | 8,10 | 32,14 | 4,83 | 33,33 | 2,71 | 21,74 | 3,04 | 53,85 | 20,56 | 63,64 | 5,29 | 25,00 | 6,75 | 25,00 | 9,33 |
| Flying aquatic adult | 4,17 | 0,81 | 3,57 | 1,00 | 8,33 | 0,40 | 4,35 | 1,14 | 7,69 | 2,59 | 0,00 | 0,00 | 0,00 | 0,00 | 25,00 | 0,46 |
| No. of fish with diet | 24,00 | | 28,00 | | 12,00 | | 23,00 | | 13,00 | | 11,00 | | 6,00 | | 8,00 | |

| October taxa | YC | | YL | | YT | | YTL | | OC | | OL | | OT | | OTL | |
| --- | --- | --- | --- | --- | --- | --- | --- | --- | --- | --- | --- | --- | --- | --- | --- | --- |
|  | O% | A% | O% | A% | O% | A% | O% | A% | O% | A% | O% | A% | O% | A% | O% | A% |
| *Lumbricus sp.* | 68,75 | 50,64 | 87,50 | 70,54 | 75,00 | 40,74 | 69,23 | 45,49 | 80,00 | 61,29 | 100,00 | 89,53 | 100,00 | 30,59 | 100,00 | 39,04 |
| Limoniidae larvae | 37,50 | 1,83 | 25,00 | 4,15 | 66,67 | 5,72 | 38,46 | 6,19 | 40,00 | 6,82 | 0,00 | 0,00 | 50,00 | 23,30 | 100,00 | 8,43 |
| Limnephilidae larvae | 56,25 | 10,70 | 62,50 | 11,73 | 66,67 | 5,56 | 30,77 | 8,56 | 60,00 | 21,41 | 0,00 | 0,00 | 0,00 | 0,00 | 100,00 | 36,82 |
| *Velia sp.* | 25,00 | 2,00 | 12,50 | 1,36 | 16,67 | 4,93 | 15,38 | 8,60 | 40,00 | 8,68 | 0,00 | 0,00 | 100,00 | 40,20 | 0,00 | 0,00 |
| Lepidoptera larvae | 12,50 | 11,36 | 12,50 | 2,47 | 25,00 | 11,61 | 0,00 | 0,00 | 0,00 | 0,00 | 0,00 | 0,00 | 50,00 | 4,58 | 50,00 | 3,34 |
| Polycentropodidae larvae | 37,50 | 3,51 | 37,50 | 0,86 | 50,00 | 5,51 | 46,15 | 2,73 | 40,00 | 0,55 | 0,00 | 0,00 | 50,00 | 0,49 | 50,00 | 0,63 |
| Tipulida larvae | 18,75 | 3,33 | 0,00 | 0,00 | 16,67 | 1,79 | 23,08 | 1,88 | 20,00 | 0,81 | 100,00 | 10,45 | 0,00 | 0,00 | 0,00 | 0,00 |
| *Simulium spp.* | 37,50 | 7,53 | 0,00 | 0,00 | 50,00 | 16,72 | 61,54 | 1,62 | 20,00 | 0,02 | 0,00 | 0,00 | 100,00 | 0,12 | 50,00 | 0,33 |
| Nemouridae larvae | 43,75 | 1,41 | 37,50 | 3,50 | 58,33 | 4,73 | 38,46 | 8,95 | 20,00 | 0,01 | 0,00 | 0,00 | 50,00 | 0,43 | 100,00 | 2,74 |
| Chironomidae* | 62,50 | 0,93 | 37,50 | 0,35 | 66,67 | 0,55 | 76,92 | 7,38 | 0,00 | 0,00 | 0,00 | 0,00 | 100,00 | 0,26 | 100,00 | 0,37 |
| Other aquatic | 31,25 | 1,52 | 37,50 | 2,83 | 41,67 | 2,10 | 38,46 | 3,41 | 40,00 | 0,04 | 0,00 | 0,00 | 50,00 | 0,03 | 50,00 | 6,23 |
| Other terrestrial | 37,50 | 5,23 | 25,00 | 2,20 | 8,33 | 0,03 | 30,77 | 4,86 | 40,00 | 0,29 | 100,00 | 0,03 | 0,00 | 0,00 | 100,00 | 2,08 |
| Flying aquatic adult | 6,25 | 0,00 | 0,00 | 0,00 | 0,00 | 0,00 | 23,08 | 0,34 | 20,00 | 0,06 | 0,00 | 0,00 | 0,00 | 0,00 | 0,00 | 0,00 |
| No. of fish with diet | 16,00 | | 8,00 | | 12,00 | | 13,00 | | 5,00 | | 2,00 | | 4,00 | | 4,00 | |
